# Species-specific behavioural responses to environmental variation as a potential species coexistence mechanism in ants

**DOI:** 10.1098/rspb.2024.0439

**Published:** 2024-08-28

**Authors:** Vanessa Menges, Merle Rohovsky, Raúl Rojas Feilke, Florian Menzel

**Affiliations:** ^1^ Institute of Organismic and Molecular Evolution, Johannes Gutenberg University, Hanns-Dieter-Hüsch-Weg 15, Mainz 55128, Germany

**Keywords:** animal personality, behavioural consistency, dominance hierarchy, environmental responses, species coexistence mechanism, storage effect

## Abstract

A fundamental question of ecology is why species coexist in the same habitat. Coexistence can be enabled through niche differentiation, mediated by trait differentiation. Here, behaviour constitutes an often-overlooked set of traits. However, behaviours such as aggression and exploration drive intra- and interspecific competition, especially so in ants, where community structure is usually shaped by aggressive interactions. We studied behavioural variation in three ant species, which often co-occur in close proximity and occupy similar dominance ranks. We analysed how intra- and allospecific aggression, exploration and foraging activity vary under field conditions, namely with temperature and over time. Behaviours were assessed for 12 colonies per species, and four times each during several months. All behavioural traits consistently differed among colonies, but also varied over time and with temperature. These temperature-dependent and seasonal responses were highly species-specific. For example, foraging activity decreased at high temperatures in *Formica rufibarbis*, but not in *Lasius niger*; over time, it declined strongly in *L. niger* but much less in *F. rufibarbis*. Our results suggest that, owing to these species-specific responses, no species is always competitively superior. Thus, environmental and temporal variation effects a dynamic dominance hierarchy among the species, facilitating coexistence via the storage effect.

## 1. Introduction

A main question of community ecology is how species coexist in the same habitat. Functional traits can mediate stabilizing and equalizing effects that allow species coexistence. Stabilizing effects are driven by niche differences between species. Equalizing effects, such as negative frequency-dependent selection, reduce average fitness differences between species and hence mitigate effects of competition for inferior species [1]. Hence, functional traits can drive competitive hierarchies [2,3]. Without stabilizing effects, however, average fitness differences inevitably lead to competitive exclusion [4,5].

Apart from morphology and physiology, behaviour constitutes an often overlooked set of traits. Behavioural traits like aggression and exploration frequently determine dominance and resource holding potential (i.e. the ability to win a fight over resources) [6–9]. In many taxa, individuals compete via direct interference competition, and the outcome of competitive contests is largely determined by aggressive behaviour. If aggression consistently varies among members of the same community, it forms stable asymmetric relationships, so-called dominance hierarchies [10,11]. Dominant individuals (or species) gain priority access to food resources, shelter or nest sites [12]. In contrast, subordinate members (or species) must find alternative strategies to cope with superior and aggressive competitors. As well as aggression, explorative behaviour is also linked to dominance [7].

**Figure 1.**
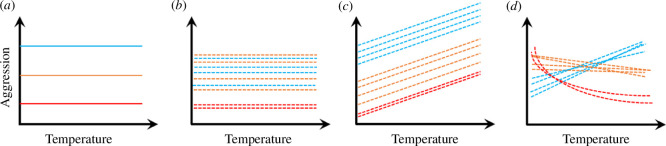
Different scenarios of environmental effects on traits. The graphs show how a trait may depend on an environmental factor in three ant species. We exemplify the scenarios with aggression as functional trait, and temperature as environmental factor, but they can be replaced by any behavioural trait that affects competitive ability, or any environmental factor such as time of year, food availability or time of day. (*a*) The conventional model of a stable dominance hierarchy, which does not consider intraspecific variation. The blue species is always dominant, followed by the orange one and the red one is most submissive. (*b*) There is intraspecific variation between individual colonies, to a degree that trait averages of the orange and the blue species are similar—they are co-dominant. This may prolong time to extinction, but does not guarantee long-term coexistence. (*c*) Aggression is temperature-dependent. Still, the dominance hierarchy remains stable. (*d*) Species differ in their responses to environmental fluctuation. As a consequence, current competitive dominance depends on the environment, since there is no species which always has the highest trait expression.

Across species, dominance hierarchies are less common, but highly important in taxa such as carnivorous mammals [13,14] or ants. Ants of different species often fight over resources, and interspecific aggression is considered key to structuring ant communities [15,16]. Dominant (territorial) and subdominant (non-territorial) species aggressively displace others from food resources, while subordinate species only defend their nest [16–18]. In certain habitats, such as tropical forest canopies, highly aggressive, dominant species even defend their territory [19], but such territorial species are absent from most other habitats, especially in the temperate zone [16]. Therefore, subdominant species form the top of the dominance hierarchy in most ant communities worldwide and shape their local ant communities [16]. Many studies investigate which stabilizing mechanisms allow lower-ranked species to coexist with dominants, including trade-offs, spatial exclusion between territorial species and niche differences [20–22]. Nevertheless, little is known about the competitive relationships between species of the same dominance rank. If they differ consistently in a trait that mediates competition, such as aggression, this should result in a stable dominance hierarchy (i.e. species A would consistently be more dominant and competitive than species B, and B consistently more competitive than C). In theory, this should lead to competitive exclusion of inferior species over time (scenario in figure 1*a*), and thus require further stabilizing or equalizing mechanisms to explain their coexistence. In this case, behavioural traits affect competition via a trait hierarchy rather than limiting similarity [2].

Behavioural variation is most often investigated *within* species. Consistent intraspecific behavioural variation, so-called animal personality traits, can act as equalizing effects because they cause within-species niche specialization, thereby reducing intraspecific competition [23,24]. If intraspecific variation is of the same magnitude as interspecific variation, it should strongly reduce interspecific competition and thus prolong time to competitive exclusion (figure 1*b*) [25]. However, intraspecific variation reduces intraspecific rather than interspecific competition and thus might not suffice to allow long-term species coexistence. However, its consequences for interspecific competition have been rarely studied until recently [26,27].

Behavioural traits were shown to vary with environmental factors such as temperature [28–30]. This should affect an individual’s fitness, but it does not promote species coexistence if all species respond similarly, even if there is intraspecific variation (figure 1*c*). This is because species can coexist only if niche differences counteract the effects of fitness differences, which in the long-term results in higher intraspecific than interspecific competition under benign conditions [31]. However, if responses to environmental variation are species-specific, fitness differences among species are counteracted when environments fluctuate (figure 1*d*) [1,5], facilitating coexistence. Such species-specific responses to environmental fluctuation form the basis of the storage effect, which represents one of the most important coexistence mechanisms to date [5]. The storage effect posits that species coexistence is promoted if species have different competitive abilities under different environmental conditions (environment–competition covariance), if this response to the environment is species-specific, and if population growth can be buffered (i.e. organisms can endure unfavourable conditions). Under favourable conditions for one species, this species will experience population growth and hence stronger intraspecific than interspecific competition, while the reverse is true for rare or declining species. Coexistence is promoted if varying environmental conditions effect that either one or the other species is favoured and at a competitive advantage. Since the introduction of this concept more than 20 years ago [31], empirical studies tested its assumptions in plants, fungi and bacteria, but to our knowledge, not in animals [32–35]. This may be because, in plants, competitive pressure and fitness is easier to approximate via traits such as seed size, inflorescence number and growth rate. In animals, competitive ability and competitive pressure are harder to measure, making it difficult to study covariance of environment and competition.

Here, ants and their behaviour form a suitable study system to investigate coexistence. Although rarely viewed as a functional trait *sensu* McGill *et al*. [36], aggression and other behaviours shape species interactions and competitive outcomes. Dominance hierarchies were often determined based on interspecific aggression patterns alone [37,38]. Since behavioural traits often covary with dominance [6], we aim to investigate whether behavioural variation have the potential to induce stabilizing and equalizing effects on coexistence in a guild of ecologically similar ants. To this end, we studied behaviour in three ant species that often co-occur in close proximity in Central European grasslands, often with nests less than 1 m apart (V.M. & F.M. 2021, personal observation). All three are highly aggressive against each other [37]. Given the absence of territorial species, their frequent co-occurrence raises the question why they do not outcompete each other, and behaviour may be key to understanding their coexistence. Therefore, we investigated how behavioural traits vary (i) over time and in response to environmental variation, (ii) across conspecific colonies, and (iii) between species.

## 2. Material and methods

### (a) Study species and study site

We examined three ant species: *Formica rufibarbis* (Formicinae)*, Lasius niger* (Formicinae) and *Tetramorium caespitum* (Myrmicinae). These species were by far the most abundant ants at the study site and many other grassland habitats nearby. *Formica rufibarbis* lives in monodomous colonies in meadows with up to 5000 individuals [39]. Individuals are 4–7.5 mm in size. *Lasius niger* is strictly monogynous and monodomous and inhabits meadows, open woodland and urban habitats. Its colonies have 14 000 individuals on average. Individuals measure 3–5 mm. *Tetramorium caespitum* is, besides *L. niger,* the most common species in Central European meadows and urban habitats. It is monodomous and monogynous, but colonies can have several 10 000 workers. Workers measure 2.5–4 mm in size. Both *L. niger* and *F. rufibarbis* have a highly plastic diet. They frequently tend trophobioses, but are also highly zoophagous (predatory and scavenging). *Tetramorium caespitum* is more granivourous, but also strongly zoophagous and can tend trophobioses. Thus, all three species have considerable dietary overlap, and often compete aggressively at food sources [37,39].

Our study site was a semi-natural, protected meadow near Mainz-Hechtsheim, Germany (49°57′22″ N, 8°17′31″ E) of approx. 12 500 m². All studied colonies were within a 50 m × 100 m rectangle; nests of several species were frequently within 1 m² (V.M., M.R., R.R.F. & F.M. [date], personal observation). Before the experiments, we marked 14 colonies per species. Independent colonies were defined as those with a minimum distance of 2 m between their nest entrances. Although individuals can forage in greater distances from their nest, nests are spatially well-defined and, in our study, never exceeded 40 cm in diameter, even if they had multiple entrances. A minimum distance of 2 m between nests was hence sufficient to distinguish two colonies, and most conspecific nests were at least 5 m apart. Previous studies report high conspecific nest densities (> 90 nests per 100 m²) both for *L. niger* and *T. caespitum* [39], which further confirms that, since nests do not overlap, they are rather small in width.

### (b) Behavioural traits

In various assays, we measured exploration, aggression towards conspecific and allospecific individuals, responses to chemical footprints, hunger and foraging activity. The traits were selected based on their high importance for competition. Aggression is probably the most important trait that confers resource-holding potential in ants [16]. Exploration represents the propensity to explore new sensory inputs, and thus potentially new food sources. Both aggression and exploration are linked to competitive dominance in various animals [6–8,40]. In ants, aggression against non-nestmates or other species is ubiquitous, but varies with the individual’s motivation and previous experience, as well as the chemical distance to the opponent [41,42] and therefore is not an all-or-nothing response. Foraging activity, measured here as number of workers at a food source, should strongly affect scramble competition, as foraging ranges of the species strongly overlap. We often observed ants exploiting food sources near the nest entrances of another species. Thus, scramble competition is important in ant communities, because more active species will exploit food resources faster and pre-empt others. Footprint following could be an indicator of intercolonial eavesdropping [43]; however, since the footprint assays turned out non-informative (see electronic supplementary material), they will be neglected here. Finally, hunger reflects a colony’s nutritional demand relative to food availability. It does not necessarily depend on colony size, but may increase when food availability declines. This will influence worker motivation to forage and compete with others. Albeit plausible, a previous study did not find a decrease in hunger after alates leave the nest in summer [30].

### (c) Experimental set-up

**Figure 2.**
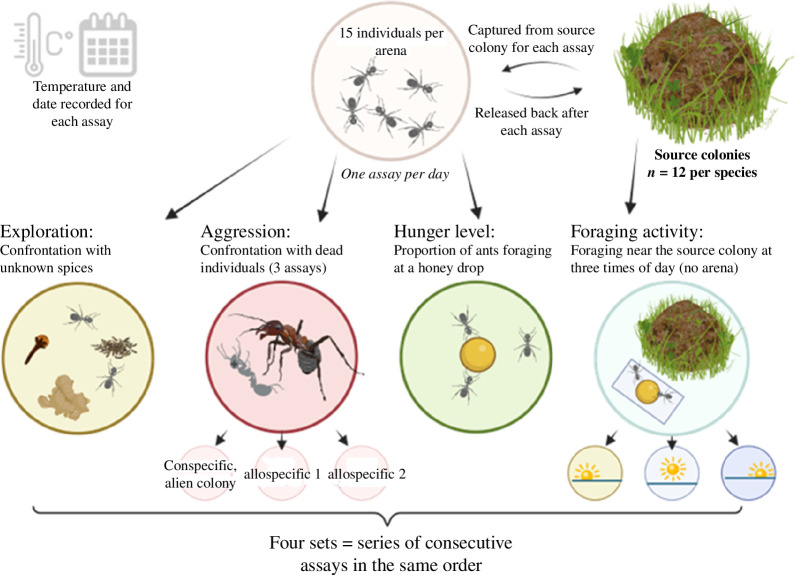
Overview of the experimental design. Ant mound drawn by Doris Franke. Image created with biorender.com.

Behavioural traits were measured in sets of nine different assays, which were repeated four times (figure 2). On a given day, we conducted only one type of assay, but measured all colonies on this day. This was to minimize and standardize potential carry-over effects of one assay to the other. The order of the different experiments remained always the same, but the order of the colonies was randomized each day. We performed the following assays in this order: exploration, response to conspecific footprints, response to allospecific footprints (against one of the three species), response to allospecific footprints (against the other of the three species), aggression against conspecifics, aggression against allospecific individuals of species 1, aggression against allospecific individuals of species 2, hunger level and foraging activity at the nest site (morning, noon, evening) (see §2d) for details). For each assay (except foraging activity at the nest), 15 ants of a colony were collected at their nest entrance and released there again thereafter, such that freshly collected ants were used every time. All experiments were directly performed at the study site in an open tent covered with UV-reflecting foil. The set-up was always standardized to the same cardinal points and filmed using 4K video camera recorders (Sony FDR-AX33). Temperature was documented with a data logger (Testo 174H, Testo SE) next to the arenas for each assay and each tested colony. Colony affiliation was blinded in the videos. There were three observers, but video analysis of each assay type was always conducted by the same observer to avoid observer effects. All assays were done between 15 June and 2 September 2021. Colonies that died during the course of the experiment were excluded from analysis if fewer than three sets were complete. Our final dataset contained 12 colonies per species. We obtained data for all four sets for all but one *Lasius* colony.

### (d) Set-up of the behavioural assays

For the aggression assays, we used a fluon-coated plastic ring as arena (diameter 12 cm, height 5 cm), with paper as substrate that was changed after each assay. We placed a fluon-coated plastic cuboid frame (5 × 5 × 5 cm) into its centre, into which the 15 ants were placed. Then, four dead workers (conspecific or allospecific, respectively, from colonies approx. 5.5 km away) were put at the four cardinal points outside the cuboid. After 2 min, the frame was lifted. We recorded all interactions towards the dead workers for 3 min. They were categorized as ‘bite’, ‘drag’, ‘antennate with open mandibles’ or ‘antennate with closed mandibles’. From this, we calculated ‘relative total aggression’, defined as the total number of biting, dragging or mandible opening events divided by the total number of interactions.

Exploration was tested by confronting 15 individuals with eight spices [44] in a similar arena. Instead of dead workers, we placed eight spices around the cuboid: anise, ginger, thyme, pepper, caraway, clove, allspice and rosemary. Once the cuboid was lifted, we recorded all antennating interactions with these novel objects for 3 min. The total number of interactions was considered as a measure of exploration. New spices were used for every experiment.

In the same arenas, we assessed foraging propensity of 15 workers, which we interpreted as hunger level. We placed a honey droplet in the cuboid and introduced the ants in the arena outside the cuboid. After 2 min, we lifted the cuboid and videotaped for 3 min. The number of ants at the honey was counted every 30 s (i.e. six times). ‘Hunger’ was calculated as the average number of ants (out of 15) drinking honey.

In contrast, foraging activity was measured directly at the nest entrances. It should depend not only on nutritional demands (hunger), but also on colony size, worker activity outside the nest and recruitment effectiveness, and hence represent a variable different from hunger. We measured activity at three different day times (8.30 CET, 13.30 CET, 18.30 CET), which represent the times of locally highest solar zenith angle ± 5 h. We placed pieces of paper (10 × 15 cm) with a honey drop in the centre at the nest entrances. After 30 min, we counted the number of ants at the drop. This metric measures activity in a foraging context; however, measuring ant activity without baits would have been hardly possible, because activity at nest entrances was often very low. Thus, measuring ant traffic would have yielded low numbers, and thus low accuracy. Moreover, many colonies possessed multiple nest entrances within 15 cm radius, whose relative traffic changed over the season.

### (e) Statistical analysis

Firstly, we analysed how each behavioural trait varied over time and with temperature, and how these effects differed between species. This was done in separate models for conspecific aggression, exploration and hunger (command *lmer,* package *lme4* [45]). For allospecific aggression, we created separate models for reciprocal aggression in each species pair, for example for *Lasius* aggression against *Formica* and vice versa. In each model, the behavioural trait in question was the response variable. Explanatory variables included *date* (in days since 1 January), *date²*, *temperature* and *temperature²*. The quadratic terms were included to allow nonlinear relationships. Each parameter was allowed to interact with *species*, which was an additional explanatory variable. Colony identity was included as random factor. Significance of each factor was assessed using Anova (package *car* [46]). We removed non-significant interactions or main effects (unless they were part of an interaction) in a step-wise manner. *p*-values were corrected for false-discovery rate [47] for each model. For activity, we included *time of day* as additional variable, which could interact with *species*. Since this model had multiple significant interactions, we then created species-specific models for easier interpretation.

Repeatability of each behaviour was analysed using the command *rpt* (package *rptR* [48]). Repeatability reflects the proportion of total variation that is reproducible among repeated measurements of the same subject or group [49]. For each trait, we used original-scale approximation; the command included repeatability estimates for *colony* and for *set*, with 1000 bootstrap replicates. We assumed Poisson distribution for exploration, hunger and activity at the nest, and ‘proportion’ for aggression (entered as number of aggressive and non-aggressive interactions).

Finally, we analysed how behaviour differed among species in a comprehensive analysis. To this end, we created a dataset with all six behavioural traits for each colony, averaged across sets. For foraging activity, we used the average of morning, noon and evening. On this dataset, we performed an NMDS ordination (command *metaMDS*) and a PERMANOVA (command *adonis2,* both package *vegan* [50]). Then, we tested whether the coordinates of the data points differed between species using linear models. The models were created separately for the two axes and assessed using Anova. To check consistency of these results, we did an analogous analysis using separate data for each colony and set (i.e. we did not average across the four sets). The NMDS coordinates were analysed using linear mixed-effects models with the coordinates of NMDS axis 1 (or 2, respectively) as response variable, *species* and *set* as explanatory variable (interaction allowed), and *colony* as random factor. All analyses were done in R 4.2.0 [51].

## 3. Results

### (a) Aggression

**Figure 3.**
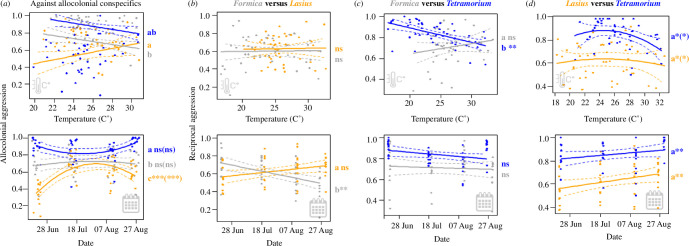
Aggression towards conspecific non-nestmates (*a*) and allospecific workers (*b–d*) for each species, depending on temperature (above) and time of year (below). The graphs show the proportions of (strongly plus weakly) aggressive interactions out of all behavioural interactions. (*a*) Aggression against allocolonial conspecifics. Data for *F. rufibarbis* are shown in grey, *L. niger* in yellow and *T. caespitum* in blue. (*b*) *Formica* aggression (grey) towards *Lasius* and *Lasius* aggression (yellow) towards *Formica*. (*c*) Aggression of *Tetramorium* (blue) versus *Formica* (grey). (*d*) Aggression of *Tetramorium* (blue) versus *Lasius* (yellow). Regression lines show estimates from linear models and confidence intervals. Letters and asterisks refer to the slopes of species-specific regression lines. Asterisks indicate whether the slope is different from zero (***p < 0.001, **p < 0.01, *p < 0.05) (asterisks for quadratic terms in parentheses if applicable). Letters refer to differences between species; slopes with same letters are not significantly different.

Temperature and date differently impacted aggression of *Lasius*, *Formica* and *Tetramorium*. Firstly, we analysed aggression towards allocolonial conspecifics (figure 3*a*). Here, date and temperature had strongly species-specific effects (significant interactions of species with temperature, date and date²; table 1). In *Lasius*, conspecific aggression increased during the season until mid-July, and then decreased (strong linear and quadratic effects of date). In *Tetramorium*, this course was roughly opposite albeit weaker. In *Formica*, date effects were non-significant (figure 3*a*). Temperature effects were weaker than date effects; aggression tended to increase with temperature in *Lasius*, but to decrease in *Formica* (marginally significant temperature effects in both species).

**Table 1.** Model results for allocolonial aggression. The table shows χ², d.f. and FDR-corrected *p*-values for the final model, and *t*, d.f. and FDR-corrected *p*-values for species-specific effects obtained from model summaries. Effects with different letters differ significantly between species. Asterisks indicate level of statistical significance: ***p < 0.001, **p < 0.01, *p < 0.05.

	χ²	d.f.	*p*	
species	60.98	2	<0.0001***	
temperature	0.20	1	0.64	
date	5.29	1	0.029*	
date²	4.51	1	0.039*	
species:temperature	7.39	2	0.034*	
species:date	43.08	2	<0.0001***	
species:date²	41.20	2	<0.0001***	
**temperature**	***t***	**d.f.**	***p***	
*Formica*	−1.98	124	0.066	a
*Lasius*	1.81	116	0.088	b
*Tetramorium*	−0.65	108	0.53	ab
**date**				
*Formica*	1.85	97.8	0.085	a
*Lasius*	5.94	95.7	<0.0001***	b
*Tetramorium*	−3.06	91.2	0.0050	c
**date²**				
*Formica*	−1.83	97.8	0.087	a
*Lasius*	−5.7	96.8	<0.0001***	b
*Tetramorium*	3.1	91.2	0.0050	c

Pairwise analysis of allospecific aggression revealed further differences in temperature and seasonal effects in the three species. Concerning reciprocal aggression of *Lasius* and *Formica*, *Formica* became less aggressive over the course of the season, while there was no season effect in *Lasius* (significant interaction species:date). Thus*, Formica* was more aggressive than *Lasius* at the start of the season, while the reverse was true at the end of the season (figure 3*b*, electronic supplementary material Table S1). Temperature did not affect aggression in this species pair.

Aggression of *Tetramorium* towards *Formica* decreased strongly with temperature. In contrast, *Formica* aggression towards *Tetramorium* remained largely constant, with a slight tendency to increase with temperature (figure 3*c*). Seasonal effects were not significant. Hence, temperature had nearly opposite effects on the two opponents here (interaction species:temperature). Finally, reciprocal aggression of *Lasius* and *Tetramorium* were largely parallel. Both decreased with temperature, but increased with date (figure 3*d*, electronic supplementary material Table S1).

### (b) Exploration

**Figure 4.**
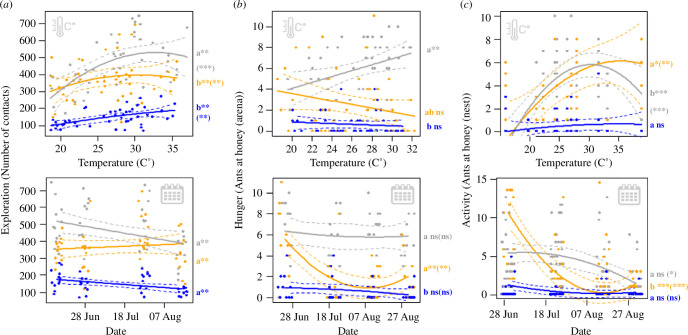
Exploration, hunger and foraging activity depending on temperature (above) and date (below). See legend of figure 3 for more details. Note that for activity, not the entire range of data points is shown for better visibility of the regression lines. Electronic supplementary material, figure S1 shows the same graphs with all data points. For foraging activity, slope comparisons stem from a comprehensive model including all species.

Exploration strongly changed with temperature and date in all species, with species-specific effects for temperature and date (figure 4*a*; table 2). Exploration increased with temperature in all species. In *Formica,* this increase was significantly stronger than in *Lasius* and *Tetramorium*, and only levelled-off at around 31°C. In contrast to the other species, *T. caespitum* only showed a weak, largely linear increase in exploration with temperature. Over the course of the season, exploration increased for all species combined.

**Table 2.** Model results for exploration (including species-specific temperature effects) and hunger (including species-specific temperature, date and date² effects). See legend of table 1 for details.

exploration	χ²	d.f.	*p*	
species	190.50	2	<0.0001***	
temperature	9.46	1	0.0051**	
temperature²	8.79	1	0.0068**	
date	12.24	1	0.0023**	
species:temperature	11.2	2	0.0076**	
**temperature**	***t***	**d.f.**	***p***	
*Formica*	4.07	112	0.00085***	a
*Lasius*	3.59	110	0.0021**	b
*Tetramorium*	3.61	110	0.0027**	b

hunger	χ²	d.f.	*p*	
species	122.09	2	<0.0001***	
temperature	0.78	1	0.38	
date	7.06	1	0.014*	
date²	6.44	1	0.016*	
species:temperature	8.83	2	0.014*	
species:date	12.04	2	0.0085**	
species:date²	11.71	2	0.0067**	
**temperature**	***t***	**d.f.**	***p***	
*Formica*	2.70	125.3	0.024*	a
*Lasius*	0.50	118.7	0.62	ab
*Tetramorium*	−1.47	124.3	0.23	b
**date**				
*Formica*	−2.02	121.8	0.083	a
*Lasius*	−3.75	106.6	0.0026**	a
*Tetramorium*	1.00	109.1	0.36	b
**date²**				
*Formica*	2.036	122.2	0.099	a
*Lasius*	3.59	106.6	0.0023**	a
*Tetramorium*	−1.06	109	0.37	b

### (c) Hunger

Ant foraging at honey in the arena showed strongly species-specific effects of date and temperature (figure 4*b*; table 2). *Lasius* foraging decreased in a quadratic way over the season and levelled off towards the end of the season, but was unaffected by temperature. In contrast, *Formica* foraging increased with temperature, but hardly changed over the season. In *Tetramorium*, hunger was neither affected by date nor temperature.

### (d) Activity

Foraging activity at the nest showed species-specific effects for most explanatory variables. For easier interpretation, we therefore ran species-specific models. In *Formica,* temperature effects were strongest: foraging activity increased until roughly 30°C and slightly decreased beyond approximately 35°C (figure 4*c*; electronic supplementary material, figure S1; table 3). In *Lasius*, activity also increased until approximately 30°C, but then levelled-off. Both linear and quadratic temperature effects were much weaker in *Lasius* than in *Formica*. Date effects showed a contrasting pattern: in *Lasius*, activity decreased from June until early August and then levelled off, whereas in *Formica,* activity decreased slower, but rather constantly, until the end of the study. In both species, activity was highest in the evening (19.30) and lowest in the morning (7.30), with noon being in between. These effects were much stronger in *Lasius* than in *Formica* (electronic supplementary material, figure S2; table 3). In *Tetramorium*, foraging activity at honey was always very low and neither affected by temperature, date, nor time of day.

**Table 3.** Results of species-specific models for foraging activity at the nest. See legend of table 1 for details.

*Formica*	χ²	d.f.	*p*
temperature	47.86	1	<0.0001***
temperature²	49.4	1	<0.0001***
time of day	9.37	2	0.013*
date	21.03	1	<0.0001***

*Lasius*			
temperature	3.65	1	0.061
temperature²	7.1	1	0.012*
time of day	26.22	2	<0.0001***
date	30.92	1	<0.0001***
date²	26.03	1	<0.0001***

*Tetramorium*			
temperature	0.93	1	0.33
time of day	2.90	2	0.35
date	2.83	1	0.28

### (e) Repeatability: consistent differences between colonies over time

Overall, aggression was the most repeatable trait in all three species, especially in *Formica* (electronic supplementary material, figure S3 and table S2). Relative total aggression against non-nestmate conspecifics was significantly repeatable in all three species. Likewise, *Formica* aggression against *Lasius* and *Tetramorium* was highly repeatable. In contrast, neither *Lasius* nor *Tetramorium* showed consistent aggression towards allospecific ants. Exploration or hunger were never repeatable in any species, with only a tendency for hunger in *Lasius*. Foraging activity was highly repeatable for *Formica* and (weaker) for *Lasius*, but not for *Tetramorium*.

### (f) Behavioural differences between species

**Figure 5.**
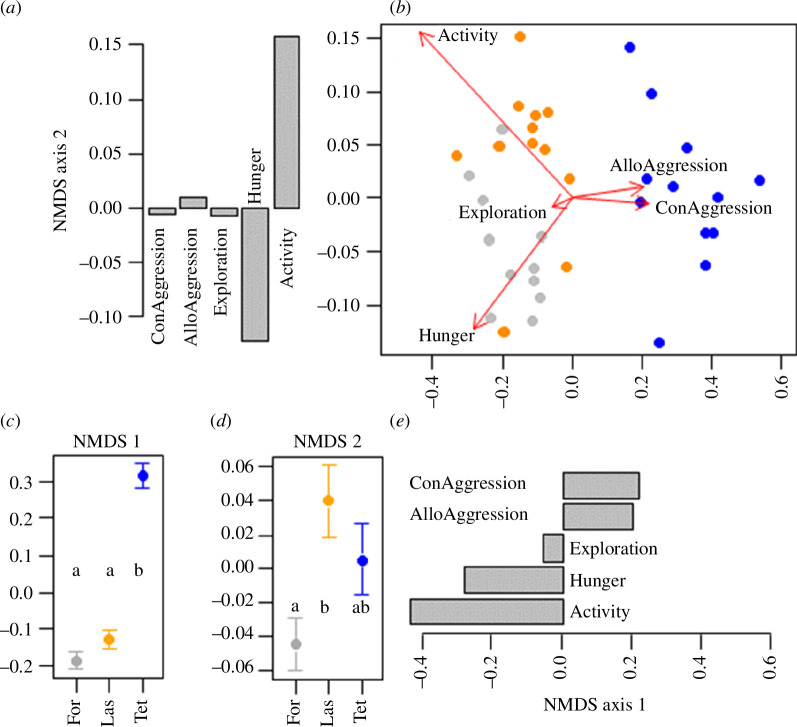
Overall behavioural variation among colonies and species. The graphs show an NMDS ordination (*b*) of all five behavioural traits for each colony, averaged across sets and associated analyses (*a,c,d,e*). (*a*) Projection of the trait coordinates on NMDS axis 2 (con aggression: conspecific aggression; allo aggression: aggression against both other species, averaged; activity: foraging activity). (*b*) NMDS ordination; each dot represents the behavioural traits of one colony, averaged across sets. (*c*) NMDS 1 coordinates and (*d*) NMDS 2 coordinates (mean ± s.e.) across species. Species with same letters are not significantly different according to linear models, followed by Tukey’s HSD. (*e*) Projection of trait coordinates on NMDS axis 1. Axes of (*a*), (*b*) and (*e*) are aligned to facilitate interpretation.

A comprehensive analysis of all behavioural traits (averaged across sets) revealed clear differences among species (PERMANOVA: *R*² = 0.89, *F*_2_ = 146.4, *p* = 0.0001; figure 5*b*). *Tetramorium*, scoring low on activity and hunger, differed most from *Lasius* and *Formica*, which scored higher in these two traits, but lower in conspecific and allospecific aggression. The coordinates of NMDS axis 1 were positively associated with con- and allospecific aggression, but negatively with hunger and activity (figure 5*e*). They were significantly higher for *Tetramorium* than for *Lasius* or *Formica* (LM: *F*_2_ = 100.97, *p *< 0.0001, figure 5*c*). NMDS axis 2 was positively associated with activity, but negatively with hunger. NMDS 2 scores differed between *Lasius* and *Formica* (*F*_2_ = 4.88, *p* = 0.014; figure 5*a,d*). Hence, *Lasius* was more active at the nest, while *Formica* foraged more in the hunger assays. This is consistent with previous analyses (figure 4*b,c*). A similar picture was obtained when the data of each set were treated separately, without calculating colony averages (electronic supplementary material, figure S4).

## 4. Discussion

This study investigated behavioural variation within and among three micro-sympatric ant species. These species often co-occur in close proximity despite little niche differentiation and occupy similar dominance ranks. This raises the question how they can coexist. We used them as a model system to test whether environmental variation can promote coexistence. In many semi-natural meadows like the study site, they represent nearly the entire ant fauna, with only few other, subordinate species of much lower abundance (F.M. 2021, personal observation). While competitively dominant ant species often co-occur with several subordinate species [17,19,38,52], the co-occurrence of aggressive, co-dominant species awaits further explanation, the more so as they do not show obvious signs of niche differentiation. Therefore, our aim was to find out whether the species form a stable dominance hierarchy, and elucidate potential mechanisms of coexistence. Owing to the important role of direct fights in interspecific contests, aggression may be the most important trait driving competitive dominance. Previous studies (albeit mostly on vertebrates) showed that behavioural traits such as aggression or exploration are linked to the dominance and resource-holding potential [6]. Our study is novel in that we study how behavioural traits vary with the environment (temperature and season) across species, while most previous studies quantified behaviour within one species only. Moreover, previous ant studies mostly studied coexistence between dominant and subordinate species, but rarely among species of similar dominance rank.

Interestingly, we could not detect a hierarchy among the three species. However, aggression and other behaviours strongly responded to environmental factors (i.e. temperature and the season) in species-specific ways. As a result, competitive dominance of each species should vary with the environment, allowing different species temporary competitive superiority (figure 1*d*). This constitutes a potential coexistence mechanism, the storage effect.

### (a) Species-specific responses to environmental fluctuations as basis for coexistence via the storage effect

All behavioural traits were influenced by either temperature or season in at least one of three species. For example, conspecific aggression increased with temperature in *Lasius*, but not in the others. Moreover, aggression of *Lasius* towards *Formica* was unaffected by temperature or date, but *Formica* became less aggressive against *Lasius* over time. This way, *Formica* was more aggressive towards *Lasius* in the early season, whereas the opposite was true later on. Analogous effects concerned temperature-dependent aggression between *Tetramorium* and *Formica* (figure 4). Hence, changing environmental conditions can lead to shifts in competitive dominance among species and hence temporally dynamic dominance hierarchies. Previous studies also found higher aggression in populations from warmer sites, compared to colder habitats [53,54]. Climate change may hence affect dominance hierarchies in unpredictable ways. Given the high ecological importance of ants in grasslands, for example as ecosystem engineers, trophobiont tenders, seed dispersers and arthropod predators [55], understanding how abiotic factors drive the behaviour of ecologically important species is crucial to protect local biodiversity.

Species-specific responses to the environment as found in this study constitute the major precondition for the storage effect as coexistence mechanism in our system. This effect is contingent on two further prerequisites [31]: for one, environment and competition should covary. This implies that abundant species experience stronger intraspecific competition than rare ones. This way, abundant species are limited by their own population size, but not by competing species. In contrast, rare species can undergo faster population growth if conditions become optimal [31,32,56]. This relation is often assumed, but to our knowledge was reported only for plants and microbes so far [32,57–59]. In animals, it is much harder to show, however, especially with relatively long-lived organisms like ants.

Finally, the storage effect requires buffered population growth. The organisms need a life stage that can persist under adverse conditions, buffering the species against unfavourable periods [56]. Ants and other eusocial insects are unique here in that they have several buffering mechanisms. In their nests, intranidal workers, brood and queen are relatively well-protected against unfavourable conditions and (to some degree) predation. Food resources are mainly ‘stored’ and distributed in the so-called ‘social stomach’ (i.e. the entirety of all colony members’ crops) [60]. Moreover, eggs and larvae can be eaten by adult ants in periods of famine, and thus represent an additional resource storage. Finally, colonies can adjust their foraging activity to environmental conditions, current competition and nutritional demands. All these mechanisms should enable ants to buffer short-term periods of hunger, whether owing to environmental conditions or owing to (currently) stronger competitors.

Although our study spanned less than three months, we believe that our conclusions—environmentally dependent behavioural variation that differs across species—are biologically relevant. Our study covers the main activity period of ant annual cycles, and, for all three species, includes the time when alate sexuals are produced and swarm [39]. During April and May, far too few ants were active outside the nests to allow behavioural experiments, and activity again declined after late August. Nests of these species are usually dormant between November and March. Hence, our evidence of species-specific environmental effects makes it likely that such patterns also exist in other phases of the ants’ life cycles.

### (b) Climatic and temporal niche differentiation

Like any form of resource partitioning, thermal or temporal niche differentiation can be a stabilizing mechanism facilitating coexistence. Here, it would be given if trait optima differed between species concerning daytime, season or temperature [22]. For foraging activity, our data do indicate temporal peaks, but they hardly differ between species, especially concerning *Formica* and *Lasius.* In both species, activity was highest in the evenings, declined over the season (albeit at different rates), and peaked around 30–35°C. However, peaks in aggression differed more between species. This includes allospecific aggression of *Formica* against *Lasius*, which decreased over time (while *Lasius* aggression did not), but also *Formica* aggression against *Tetramorium*, which increased with temperature, while *Tetramorium* aggression did not. Exploration in *Formica* increased in a quadratic way with temperature, while temperature-driven increases in *Lasius* and *Tetramorium* were significantly lower. This suggests that there is climatic niche differentiation in our system, but it is more subtle than expected. Although range and optimum of the foraging activity niches did not differ between species (unlike communities studied by [22,61]), aggression traits did, which probably translate to competitive dominance.

Effects of temperature on behaviour were reported in laboratory studies, albeit not in an interspecific context. For example, ants from warmer regions were more aggressive, but less explorative than individuals from cooler areas [54]. Segev *et al*. [28,29] reported effects of current temperatures on certain behaviours, while the climate of the collection site was hardly influential. In this context, weather conditions during a study can drive detected patterns. In a study on *L. niger* on the same site as ours, but 1 year earlier (2020), hunger increased over time [30], while it decreased in the present study (2021). Compared with 2021, 2020 was warmer in July and August, and had considerably less rainfall from June to early August (electronic supplementary material, figure S5). The drier conditions in 2020 probably caused a food shortage. This explains the higher attractiveness of baits at the nest, and the increase in hunger over time in 2020, which did not happen in 2021. Hence, climate change may alter ant behaviour and competitive interactions also by altering resource availability and thus nutritional status of ants.

### (c) Trade-offs

Trade-offs between dominance and another trait are often reported as stabilizing effects facilitating coexistence of between dominant (or subdominant) and subordinate species. This includes dominance–thermal tolerance trade-offs [61,62], which basically result in partitioning of the realized temporal niche, and dominance–discovery trade-offs, which represent differences in foraging strategies [22,63]. Neither of these trade-offs could be confirmed here. Also within species, no behavioural syndrome or trade-off was apparent. Hence, we found no evidence that intraspecific behavioural variation mediates intra- or interspecific coexistence in our system.

For foraging-related traits such as hunger, activity and exploration, *Tetramorium* usually ranked lowest. This may be owing to *Tetramorium* being more granivorous than *Lasius* and *Formica* [39], therefore being less attracted to the honey used in some assays. Simultaneously, *Tetramorium* often ranked highest in conspecific and allospecific aggression [39]. These two features set *Tetramorium* clearly apart (figure 5, electronic supplementary material, figure S4); however, this is more likely owing to dietary differences than an actual trade-off. Note that a dominance–discovery trade-off would be a highly plausible conclusion if we did not know the species’ diet, exemplifying the difficulty to design behavioural assays suitable for multiple species.

### (d) Variation among colonies—animal personality traits

Next to interspecific differences, all three species expressed consistent intraspecific variation, especially concerning aggression (electronic supplementary material, figure S3). Behavioural differentiation can contribute to individual niche specialization, reducing intraspecific competition and promoting intraspecific coexistence [23,26,30], for example if boldness differences lead to inter-individual differences in space use [27]. However, stable coexistence among species requires that intraspecific competition is stronger than interspecific competition under favourable conditions [31]. Personality differences may lead to the opposite effect, mediating fitness advantages to a behaviourally variable species but not necessarily its coexistence with others. Thus, personality differences cause equalizing rather than stabilizing effects (figure 1*b*). The same is true for genetic intraspecific variation, which may promote coexistence of conspecific colonies [31], but not necessarily its coexistence with other species. Here, genetic and plastic effects on colony-level behaviour are hard to disentangle, especially since single workers can strongly influence colony-level behaviour [64].

## 5. Conclusion

Behavioural traits vary within and across species and may be key for competitive dominance, i.e. the ability to monopolize and exploit food resources [6,7,40]. We showed that a fluctuating environment affects ant behaviour in species-specific ways, such that their competitive ability is likely to fluctuate with the environment. This leads to dynamic dominance hierarchies, suggesting that the storage effect, a fluctuation-dependent mechanism, may contribute to species coexistence in our system. There was surprisingly little evidence for niche differences or trade-offs among the species, which is consistent with previous studies [21,22]. We propose that generally, coexistence is not only facilitated by strong niche differences like different niche optima, but can also be promoted by subtle differences, such as different shapes of trait–environment curves, which alter competitive dominance depending on the environment [61]. Such differences would probably go unnoticed in most experimental approaches, but may be an underappreciated key to understanding coexistence. This may explain coexistence of groups of ecologically similar species groups without obvious niche differences [65].

Being relatively sessile and long lived, ants are a good model system to study coexistence mechanisms and the impact of interacting neighbours on short- and long-term fitness. Their unique life history allows assessing multiple short- and long-term fitness proxies and bridge the gap in our understanding of plant and animal ecological strategies [66].

## Data Availability

All data used in this manuscript are submitted as electronic supplementary material. This includes a data sheet as text file, the R script and a README file. Supplementary material is available online [67].
